# The House of Steinway

**Published:** 1875-04

**Authors:** 


### THE HOUSE OF STEINWAY.

The honors conferred upon the House of Steinway on account of the perfection of its pianos, have been almost too numerous to recount. Kings and queens and many titled personages have been numbered among its cash customers. Its fame is as widespread as the science of music itself.

Perhaps the most grateful and important recognition of the superiority of the Steinway piano which has occurred, was the spontaneous regret expressed by the musical committee at the absence of instruments manufactured by this house at the Great Vienna Exposition, in 1873. The committee, composed of fourteen of the most eminent musical experts of Europe, unanimously said: "It is much to be deplored that the celebrated inaugurators of the new system in piano-making, Messrs. Steinway & Sons, of New York, to whom the entire art of piano-making is so greatly indebted, have not exhibited."

It was admitted by all who carefully examined this department of the Great Exposition, that no instruments were exhibited which could be considered equal in point of volume and sweetness of tone to the ordinary pianos of the house of Steinway. At the Paris Exposition, in 1867, the highest Gold Medal of Honor was awarded to these instruments, and the report of the distinguished jury was full of the highest encomiums upon them. After describing the Steinway Overstrung System in Grand Pianos, the valuable improvements introduced into the Upright Pianos by Messrs. Steinway, especially their Patent Resonator, the report concludes as follows:—

"From what has been said, it may be inferred that the large tone of pianos is a true acquisition to art—an acquisition, the results of which may be increased by future improvements, and the great merit of which can not be doubted except by settled prejudice. The pianos of Messrs. Steinway & Sons are endowed with the splendid sonority, and that seizing largeness and volume of tone hitherto unknown, which fills the greatest space. Brilliant in the treble, singing in the middle, and formidable in the bass, this sonority acts with irrresistible power on the organs of hearing. In regard to expression, delicate shading, variety of accentuation, the instruments of Messrs. Steinway have over those of their competitors an advantage which can not be contested. The pianist feels under his hands an action pliant and easy, which permits him at will to be powerful or light, vehement or graceful. These pianos are, at the same time, the instrument of the virtuoso who wishes to astonish by the eclat of his execution, and of the artist who applies his talent to the music of thought and sentiment bequeathed to us by the illustrious masters. In one word, they are, at the same time, the pianos for the concert-room and the parlor, possessing an exceptional sonority."

Since commencing business in this city, in the year 1853, the Steinways have manufactured 35,000 pianofortes, every one of which has given perfect satisfaction to the purchaser. The manufactory is now said to be not only the most perfectly arranged, but at the same time the most extensive establishment of its kind in the world, the official Internal Revenue returns as published for the years 1866, 1867, 1868, 1869, and 1870, having revealed the startling fact that the number of pianos sold by this house, and the amount of its sales, exceeded those of the twelve next largest piano-manufacturers of New York combined.

---

Old People should be Careful.—An old man is like an old wagon; with light loading and careful usage it will last for years; but one heavy load or sudden strain will break it down and ruin it forever. Many people will reach the age of fifty, sixty, or even seventy years, measurably free from most of the pains and infirmities of age, cheery in heart, and sound in health, ripe in wisdom and experience, with sympathies mellowed by age, and with reasonable prospects and opportunities for continued usefulness in the world for a considerable time. Let such persons be thankful, but let them also be careful. An old constitution is like an old bone—broken with ease, mended with difficulty. A young tree bends to the gale, an old one snaps and falls before the blast. A single hard lift; an hour of heating work; a run to catch a departing train; an evening of exposure to rain or damp; a severe chill; an excess of food; the unusual indulgence of any appetite or passion; a sudden fit of anger; an improper dose of medicine: any of these, or other similar things, may cut off a valuable life in an hour, and leave the fair hopes of usefulness and enjoyment but a shapeless wreck.

---

Love gives itself, but is not bought.

